# Diagnosis and management of shrimp allergy

**DOI:** 10.3389/falgy.2024.1456999

**Published:** 2024-10-18

**Authors:** Bin Brenda Su, Warren Blackmon, Chun Xu, Christopher Holt, Nathaniel Boateng, Darren Wang, Vibha Szafron, Aikaterini Anagnostou, Sara Anvari, Carla M. Davis

**Affiliations:** ^1^ Immunology, Allergy, and Retrovirology Division of the Department of Pediatrics at Baylor College of Medicine, William T. Shearer Center for Human Immunobiology, Texas Children’s Hospital, Houston, TX, United States; ^2^ Department of Health and Biomedical Sciences at the University of Texas Rio Grande Valley, One West University Blvd, Brownsville, TX, United States; ^3^ Department of Biosciences, Rice University, Houston, TX, United States

**Keywords:** shrimp allergy, genetics, environmental exposure, diagnosis, management, specific IgE

## Abstract

Shrimp allergy, the most common food allergy in the United States, affects up to 2% of the population. Its etiology is multi-factorial with the combination of genetic predisposition and environmental exposures. This review summarizes the latest diagnosis and management strategies for shrimp allergy. Currently, the double-blind, placebo-controlled food challenge is the gold standard for diagnosis. Moreover, mainstream and experimental management strategies include food allergen avoidance, the FDA-approved omalizumab, and oral immunotherapy. Herein, we emphasize the urgent need to develop more effective diagnostic tools and therapies for shrimp allergy.

## 1 Introduction

### 1.1 Epidemiology

Food allergy (FA) involves complex immune responses to proteins and in some cases, carbohydrates, present in specific foods, approximately 10% of the world's population suffers from food allergies (1, 2). The exact prevalence rates depend on the method of diagnosis, geographic areas, and consumption habits. The prevalence of shellfish allergy has been reported as up to 10.3% and is observed in many parts of the globe (3). Pediatric shellfish allergy increased from 0.5% in 2004 to 1.3% in 2019 in the US (3, 4). Shrimp allergy (SA) is the second most common FA in the United States, affecting up to 1.3% of the population (2, 5). There are some variables of shellfish and shrimp allergy in different regions and ages that are summarized in Table 1 (2, 6–17). The possible symptoms of shrimp allergy range from mild to moderate, to severe, and even life-threatening anaphylaxis, accidental exposure to shrimp is a major cause of visits to the emergency room, with anaphylaxis occurring in up to 50% of those with shrimp allergy (2, 18, 19).

**Table 1:** The prevalence of shellfish, shrimp—area and age.

Continent	Shrimp				Shellfish			
	Region/Country/City	Prevalence%	Age	Refs	Region/Country/City	Prevalence%	Age	Refs
Asia	South Asia	3.4	3–7 yrs^a^	Lao-araya et al. (6)	Hong Kong, Philippines, Singapore	0.9–1.3	<7 yrs	Leung et al. (13)
	China	4.4	Preschool	Zeng et al. (7)		5.1–7.7	Adolescents, adult	
	Hong Kong, China	0.1	NA	Li et al. (8)	Singapore	1.2 (4–6 yrs); 5.23 (14–16 yrs)		Shek et al. (14)
	Guangzhou, China	0.18			Philippines	5.12	14–16 yrs	
	Shaoguan, China	0.7			Guangzhou, China	5.1	School children	Yang et al. (15)
	India	15.5^b^; 3.2^c^	Adult	Magesh et al. (9)	Shaoguan, China	1.8		
Europe	Northern, Central, Southern Italy	4, 13, 16	NA	Asero et al. (10)	Michigan	16	College students	Greenhawt et al. (16)
	Denmark	2		Ostertale et al. (11)	France	0–10.3	NA	Moonesinghe et al. (17)
Northern America	U.S.A	1.9	2.5% at 30–39^d^	Guputa et al. (2)		5.5	5–17 yrs	
Africa	Ghana	0.1	5–16 yrs	Obeng et al. (12)	U.S.A. (12)	2.9	3.6% at 30–39^d^	Gupta et al. (2), Wang et al. (3)
Worldwide		0.3–0.6	NA	Wood et al. (20)		9	Adult	

^a^ yrs: years old.

^b^ overall weighted population.

^c^ weighted population if total IgE <median.

^d^ highest age with allergy between 18 and 60 yrs old.

### 1.2 Shrimp allergens

Shrimp is popularly consumed shellfish because of its rich nutritional value, and particular protein content. However, many shrimp proteins (allergens) can cause allergic reactions in some people. Currently, a total of ten shrimp allergens have been registered based on the World Health Organization and International Union of Immunological Societies’ Database (21, 22), including the major shrimp allergen tropomyosin (TM) and the minor allergens including arginine kinase, myosin light chain, sarcoplasmic calcium-binding protein, troponin C, hemocyanin, triosephosphate isomerase, fatty-acid-binding protein, and glycogen phosphorylase, plus two potential allergens, enolase, and aldolase (22). A few more potential shrimp allergens have been revealed: shock protein 70, α-tubulin, chymotrypsin, β-enolase, Eno, aldolase A, glyceraldehyde-3-phosphate dehydrogenase, and cyclophilin (22).

Shrimp TM is a ubiquitous structure muscle protein and is a cross-reactive invertebrate pan allergen because of a high homology with the TM from dust mites and cockroaches (23–28). Shrimp tropomyosin (Pen a 1), the muscle protein invertebrate pan-allergen functions in muscle contraction, and heat-stable (29, 30). However, there are conflicting reports on the IgE-binding to heat-stable allergens, such as TM and myosin light chain (31). The conflicting results of heat-treatment may reveal the importance of shrimp species and environmental influences on patient IgE profiles in determining allergenicity. Evidence has shown persistent certain allergenicity from shrimp tropomyosin under low pH conditions because of the conservation of its linear epitopes (32). This emphasizes the role of solubility and the isoelectric point of proteins in allergenicity.

A strong correlation between shellfish and HDM sensitization, most likely because there are 81% homologs of TM in amino acid sequence similarity between prawns and HDMs and 82% similarity between prawns and cockroach (33–36). The sequence identity of the HDM TM (Der p 10 and Blot 10) to the identified eight IgE epitopes of Pen a 1 was >80% (36, 37). More studies have indicated the association of exposure to house dust mites or cockroaches with peanut and shrimp allergy (38–40). Therefore, the IgE cross-reactivity with shellfish tropomyosins accounts for mild oral allergies when people consume shellfish (36).

### 1.3 Etiology and pathogenesis of shrimp allergy

Shrimp allergy, like other complicated diseases, is caused by several factors, including genetic and environmental exposures. Evidence shows that genetic and environmental factors are involved in shrimp allergy development, even though the exact causes of shrimp allergy are not fully understood (41–53).

### 1.4 Genetics in shrimp allergy

Genetics plays a crucial role in the manifestation of food allergy (41). Compared to fraternal twins, there exists a greater probability that identical twins are both allergic to peanuts, indicating that peanut allergy is at least partially heritable (42). Kivisto et al., (2019) found a similar trend for pistachio, walnut, sesame, and fish. Several associations of gene mutations, variants, and single nuclear polymorphisms have been found in genes associated with food allergy, but not necessarily as causative factors (43). For example, filaggrin (FLG) is a general food allergy risk gene, HLA has food-specific effects, and mucosa-associated lymphoid tissue lymphoma translocation 1 (MALT1) variants increase the risk of sensitization and the development of allergy (44). A few reports have unveiled specific genetic markers linked to an elevated risk of developing shrimp allergy (Table 2). These shrimp allergy-associated genes include HLA-DQ (rs9275596), HLA-DRB1 (HLA-DRB1*04:05-HLA-DQB1*04:01), IL-13 (rs20541, and IL13 rs1800925) (45, 46), and food allergy -associated gene TLR4 rs4986790 (Asp299Gly) (47). Genome-wide association studies identified the causal effect of SA on the occurrence of major depressive disorder (48). All these studies suggested a strong association between genetic factors, and three out of four studies listed in Table 2 investigated shrimp allergy (Table 2).

**Table 2:** Summary of the studies of host genetic susceptibility for shrimp allergy.

	Sample size	Populations	Genes Identified	Findings	Author
1	130 infants/110 controls	Turkish	rs1898830 & rs5743708—TLR2	rs4986790 (Asp299Gly) in TLR4 gene associated with children with food allergies.	(47)
			rs4986790 & rs4986791—TLR4		
2	30,206 participants with shrimp allergy based on two sample sets	East Asian population	44 shared by Shrimp Allergy (SA) and Major Depressive Disorder (MDD) at the genomic level; 17 shared by SA and MDD in brain tissue; 6 genes shared by SA and MDD in blood samples	GWA identified the causal effect of shrimp allergy on the occurrence of MDD,	(48)
3	532 participants	India	HLA-DQ rs9275596,	HLA-DQ and IL13 polymorphisms pose a major risk for shrimp-allergic patients	(45)
			IL13 rs20541, and IL13 rs1800925		
4	11,011 participants	Japanese	HLA-DRB1*04:05-HLA-DQB1*04:01	Using GWAS, HLA-DRB1*04:05-HLA-DQB1*04:01 associated with shrimp allergy	(46)

*Represents the allele of this gene.

Although current genetic studies have low statistical power and there is relatively small sample size as well as heterogeneity in the definition of shrimp allergy, studies reveal several genetic loci/genes, which implicate the importance of barrier and immune function genes in shrimp allergy. Furthermore, variations in genes responsible for the breakdown and metabolism of specific proteins in shrimp can impact an individual's susceptibility to allergic reactions. Integrative approaches, including genetics/genomics with transcriptomics, proteomics, and metabolomics, will be critical next steps to translating these genetic insights into practice. The biggest challenge in shrimp allergy genetics is elucidating specific mechanisms of action for shrimp allergy risk and pathogenesis for the loci.

The knowledge of the genetic underpinnings of shrimp allergy holds significant implications for diagnosis. Genetic testing aids in identifying individuals allowing for early interventions at higher risk individuals. In the future, by applying single nucleotide polymorphism (SNP) genotyping through whole genome amplification (WGA), candidate gene study (e.g., human leukocyte antigen, HLA region), whole exome sequencing (WES), or whole genome sequencing (WGS) of shrimp allergy patients, we can better identify clinical risk factors and genetic loci associated with shrimp allergy during screening to reduce its incidence. The genetic findings in shrimp allergy may shed light on the contribution of human genetics to the susceptibility to shrimp allergy.

### 1.5 Environmental exposures

Several environmental factors are involved in the pathogenesis of shrimp allergy development. A few hypotheses associated with shrimp allergy development with environmental variables such as mono-allergen and dual-allergens exposure, dietary, hygiene, intestinal microbiota, vitamin D, pollution, co-exposure to dust mites or cockroaches, and microbes (49–56). Here we discuss a few environmental exposures.

The hygiene hypothesis suggests that a post-natal environment lacking sufficient exposure to allergens and pathogens could lead to an undeveloped immune system or misdirection of the immune system towards otherwise tolerated allergens (49). Lynch and colleagues found that in the first year of life, “exposure to cockroach, mouse and cat allergens was negatively associated with recurrent wheeze (*p* < 0.01)” (50). The intestinal microbiota forms a barrier that may promote or suppress food allergies (51). For example, *Dorea* may reduce the allergic risk of shellfish, while *Ralstonia* may promote it and *Bacillus coagulans*, *Bifidobacterium infantis*, and *Bifidobacterium lactis* have been utilized as potential therapies (50, 52, 53).

This phenomenon extends beyond topical or respiratory allergens. Until 2008, the American Academy of Pediatrics recommended avoiding allergenic foods until 3 years of age, and the incidence of peanut and other allergies increased exponentially (53). A team led by Professor Gideon Lack of King's College in London, along with the Immune Tolerance Network and Food Allergy Research & Education, launched the Learning Early About Peanut Allergy study to probe whether early introduction of peanuts would be effective in preventing the development of peanut allergy in high-risk children. They found that introducing high-risk children to peanut foods early was associated with more than an 80% reduction in developing peanut allergy (54). This study showed young children's immune systems exhibit a high degree of plasticity and require some level of exposure to food allergens during early life to be able to modulate immune responses that can persist for the rest of their lives. The cause of this phenomenon is likely multi-factorial (55, 56).

Together, genetic susceptibility and environmental factors determine the complex etiology and pathogenesis of shrimp allergy.

## 2 Diagnosis of shrimp allergy

The diagnosis of shrimp allergy is based on history, skin prick test (SPT), and sIgE to shrimp allergens, as recommended by the guidelines for diagnosis and management of food allergy in the United States, and the European Academy of Allergy and Clinical Immunology (EAACI) guidelines for IgE-mediated food allergy (57). The important step of the diagnostic process involves reviewing the patient's history to identify any known reactions to shellfish. If a reaction has occurred recently, there is a higher risk of current allergy. When patients ingest TM, it may cause a Type II-mediated immunological reaction, manifesting in symptoms such as urticaria, flushing or urticarial rashes on the face and/or extremities, localized itching, and nausea. The patient may experience severe reactions, including vomiting, difficulty breathing, widespread urticarial rashes, swelling (angioedema) of the mouth and throat, or anaphylaxis (58).

The administration of a SPT entails puncturing the skin to deliver a food allergen. A reaction to the allergen is characterized by wheal and rash around the area of application. Skin prick testing offers fast results at a relatively low cost and is known to have high negative accuracy, in which a lack of response is generally attributed to a lack of allergy. However, the variable protein concentrations of the food allergens in extracts and fresh food may lead to inconsistent results for these foods (59).

ImmunoCAP^TM^, a fluorescence enzyme immunoassay, is a useful tool in the diagnostics of shellfish allergy to measure the serum-specific IgE (sIgE) against whole food (f24, shrimp allergens), including shrimp tropomyosin (f351 rPen a 1, TM), including TM or other minor allergens. Shrimp specific IgE binding proteins, like other allergens, can be identified by Western blotting and mass spectrometry (60–63). Specific IgE diagnostic tools are very useful but cannot unilaterally determine shrimp food allergy reactivity. Patients with a clinical history of mild reactions to shellfish may exhibit high levels of tropomyosin sIgE, while others with a history of severe anaphylaxis may have low or even undetectable levels with conventional assays (64, 65). Dust mite and cockroach sensitization can cause positive testing due to homology and IgE cross-reactivity.

The gold standard for diagnosing food allergy remains the double-blind, placebo-controlled food challenge (DBPCFC) (66) using oral food allergens. This allows clinicians to confirm or deny a patient's stated history within a controlled environment and administration of known concentrations of whole allergen. Titration upward of the allergen administration beginning with protein levels far below what would be found in the actual food allows for significantly safer evaluations of patients even with severe shellfish allergy.

There are also some experimental diagnostic tests in development. Basophil activation tests (BAT) measure the degree of degranulation and activation of basophils caused by the cross-linking of IgE to the FcɛRI. This test utilizes flow cytometry to analyze specific cell surface markers (including CD63) presenting on the activated basophils. It provides insight into the patient's expected degree of reactivity (67). This test has had mixed results, with basophils being short-lived and present in low concentrations or absent in 10%−15% of patients, it can be falsely negative, especially immediately post allergic reaction. Recently, a novel test has emerged, IgE-crosslinking-induced luciferase expression. A complete set of FcɛRI subunits was transfected into a rat basophilic leukemia-derived mast cell line and sensitized with patient sera. This test was shown to have lower accuracy than shrimp extract-based BAT (64, 68) but holds the potential for testing specific epitopes and peptide segments of shrimp allergens, allowing for further elucidation of the specific mechanisms behind an individual's allergy. The mast cell activation test is also a sensitive test for peanut allergy (67). Component-resolved diagnosis by Western blot, Enzyme-Linked Immunosorbent Assays (ELISA), light-initiated chemiluminescent assay, mass spectroscopy, and bead-based epitope assays all suggest that TM has a higher diagnostic value than shrimp extract (64).

In summary, currently, diagnostic tools for food allergy including shrimp allergy remain unsatisfactory, with a shared decision-making process for use recommended by experts in the field (64). In the future, combined genetic, epigenetic, skin prick tests, and blood tests with the family history and presence of atopic co-morbidities of patients will further refine our ability to diagnose and predict the development of shrimp and other food allergies. Consideration of other samples such as feces, saliva, and urine for other novel tests and metabolic biomarkers may be utilized in the future. More accurate, earlier, and sensitive diagnosis tools are urgently needed.

## 3 Food allergy prevention

Education is critical for shrimp allergy prevention. For example, the knowledge of reading labels to limit accidental exposure to allergenic foods containing shrimp and other shellfish, as well as educating parents on the early introduction of a variety of foods gradually to their children is essential for prevention of allergic reactions (66). Proper vitamin D intake is helpful, as deficiency is associated with food IgE sensitization (69).

Smeekens et al., revealed that the vaccination with shellfish allergen DNA using a PowderJect XR DNA vaccine delivery system increased shrimp-specific IgG and C3H/HeJ is the best among three strains of mice (70). Kubo et al. reported that a single DNA plasmid vaccine constructed from Litopenaeus vannamei (Lit)-lysosomal-associated membrane protein (lit-LAMP-DNA-vaccine) promoted Th1 responses, thwarting anaphylaxis in shrimp-sensitized mice, suggested the lit-LAMP-DNA-vaccine can be developed to prevent or treat shrimp allergy (69–71). Wai et al., showed two hypoallergenic TM molecules could reduce IgE reactivity and allergenicity and induce blocking IgG antibodies in humans as a proof of concept for peptide vaccination (71). Overall, given the difficulty of developing clinical trials for vaccines to patients at risk for anaphylaxis, it is unclear how long and how safe and efficacy of these successful vaccines in mouse models will translate clinically to humans. Therefore, other interventions for effective prevention strategies with less or no advert event and stress for shrimp allergy urgently need to be developed.

## 4 Management of shrimp allergy

### 4.1 Conventional management

The management of shrimp allergy is based on the guidelines for diagnosing and managing food allergy in the United States (72). In practice, the key current management for shrimp allergy is to avoid eating shrimp. A strict avoidance or an elimination diet is the best way to prevent any allergic reaction to food. Hence, it is imperative to educate parents and pediatric and adult patients with food allergies on how to avoid consuming allergenic foods.

In addressing shrimp allergy, researchers aim to decrease IgE expression, increase blocking IgG expression, and prevent pro-inflammatory responses. Shrimp oral immunotherapy (OIT) (73, 74) has been shown to be successful in case series with and without omalizumab co-treatment. Sublingual immunotherapy for shrimp also improved the clinical symptoms for shrimp allergic patients who have house dust mite allergy (75). Refaat et al. (2014) administered shrimp sublingually to shrimp-allergic patients and observed lower specific-IgE (76). Most recently Theodoropoulou and colleagues reported that sublingual immunotherapy exhibited safe and effective desensitization to shrimp (74). Oral food Challege (OFC) is the gold standard for diagnosing a food allergy, including a shrimp allergy, and practitioners should follow standardized protocols to achieve consistent outcomes.

On February 16, 2024, following the success of the OUtMATCH study, the FDA approved omalizumab, the first drug treatment for adults and children >1 year with food allergies, including shrimp-allergic patients. Omalizumab is an injectable monoclonal antibody (mAb) drug that binds to and neutralizes IgE, the human antibody that mediates allergic reactions. Initially approved for chronic spontaneous urticaria, omalizumab has proved efficacious in the management of multiple food allergies and functions best when paired with OIT and an avoidance diet (77). However, the primary adverse effects of OIT and omalizumab, separately, include life-threatening anaphylaxis in 1.65–10.9% and 0.2% of treated patients, respectively (78).

### 4.2 Experimental management

Many promising treatments are on the horizon for the treatment of food allergies, including other monoclonal antibodies. Tezspire (tezepelumab) is currently the only approved thymic stromal lymphopoietin (TSLP) inhibitor for asthma and is also under evaluation as a potential candidate for food allergies. TSLP is a protein that binds to many pro-inflammatory cells and is stimulated by a variety of inducers (79). Etokimab is a monoclonal antibody (mAb) that allosterically inhibits IL−33, a pleiotropic cytokine that regulates many immune responses, including those in inflammatory allergic reactions (80). Dupilimab, also a mAb, allosterically inhibits IL4 and IL13, two TH2 cytokines that are implicated in allergy and IgE-mediated immune responses (81). However, all these potential therapies remain in the experimental stage. This is the same for probiotics, herbal medicine, and other vaccination strategies which are all being explored as potential therapies (82–85). Recently shrimp allergy animal models are allowing for the development of better diagnostic and therapeutic tools (85–90).

In summary, the conventional management for shrimp allergy includes avoiding eating allergic foods, OIT, and omalizumab. Vaccination is an emerging treatment that may be developed in the future (Table 3). More effective and less risky management strategies are urgently needed for shrimp allergy.

**Table 3:** Shrimp allergy management.

		Management Strategy	Description	Advantages	Limitations	References
1	Mainstream	Allergic food avoidance	Know the specific foods that trigger an allergic reaction and avoid consumption of these foods. Educate yourself, inform others, and understand how to read food labels.	The best way to prevent an allergic reaction	Can be hidden or unlabeled ingredients	(72)
2		Oral immunotherapy	Oral immunotherapy can be used to desensitize food allergy. One consumes increasingly small amounts to bolster resistance then maintains a daily dosage.	Potential for long-term protection or toleration of small doses and improved quality of life	Risk of adverse reaction and significant commitment required	(73, 74)
3		Omalizumab	Monoclonal antibodies can target IgE to elicit immune response. Omalizumab is a new injectable therapy that has shown potential in increasing reaction threshold for peanut and other food allergies.	Increased tolerance of food allergen and reduced anxiety for allergic reaction	Potential high costs and/or side effects	(77)
4		Herbal medicine	It is based on the traditional Chinese medicine formula WuMei Pill and is aimed to improve the body's immune system.	Natural and personalized approach, cost-effective	Not standardized and less accepted. It is hard to generalize as it is a personalized and precious medicine.	(82–84)
5		Vaccination strategies	Various potential vaccination strategies to protect from food allergy are being researched, including allergen-specific immunotherapy. This includes DNA vaccines that can build tolerance.	Potential for widespread availability and long-term management	May be me with more hesitancy and variance among individual responses	(69–71)

## 5 Conclusion

**Figure 1:**
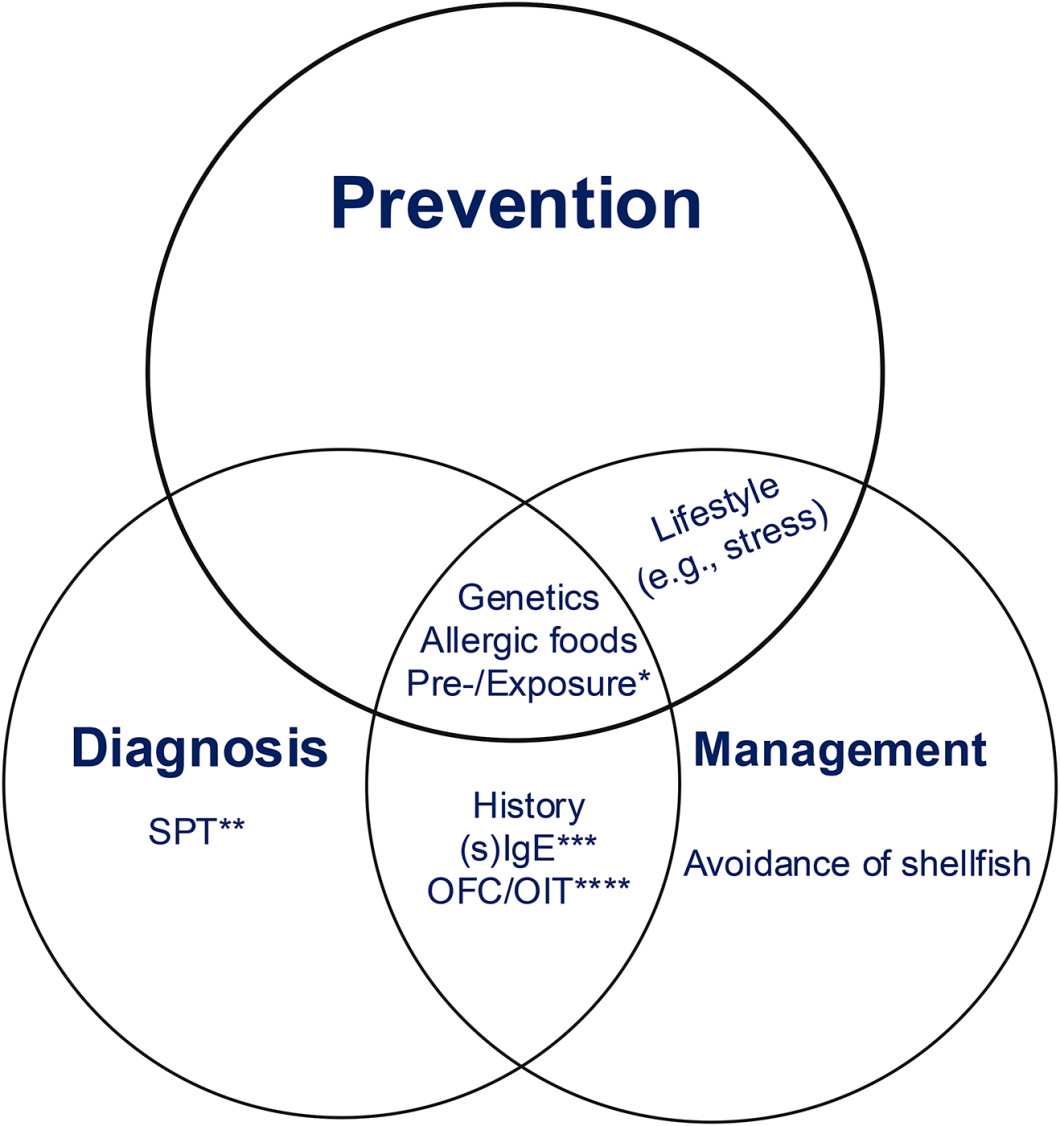
Diagnosis, prevention, and treatment of Shrimp allergy. Genetic predisposition, pre- or/and exposure, and dietary choice may be three important points to consider for diagnosing, preventing, and managing shrimp allergy. Prevention is the most important among these three. The lifestyle, such as stress or stable emotion, is for prevention and management, OFC and sIgE are for diagnosis and management. *Pre-exposure or and exposure to microbes, toxins, or other allergens. **Skin prick test. ***Specific IgE for diagnosis and monoclonal antibody against humanimmunoglobulin E (e.g., omalizumab) for management. ****Specific food allergens-challenge for Diagnosis and specific food allergens-oral immunotherapy for Management.

The intricate relationship between genetic predisposition and environmental exposures to cross-reactive allergens and food allergens highlights the personalized nature of food allergic responses. Genetic markers associated with the immune system and environmental and food exposures significantly contribute to an individual's susceptibility, severity, and onset of shrimp allergy and food allergy in general (Figure 1). This knowledge not only enhances our understanding of the underlying mechanisms but also has practical implications for diagnosis and personalized treatment strategies, ultimately improving the management of shrimp allergy for affected individuals. There is a critical need to develop more effective diagnostic tools, and effective treatments for the millions of patients who suffer from life-threatening shrimp allergy.

## References

[1] Commins SP James HR Stevens W Pochan SL Land MH King C Delayed clinical and ex vivo response to mammalian meat in patients with IgE to galactose-alpha-1,3-galactose. J Allergy Clin Immunol. (2014) 134(1):108–15. 10.1016/j.jaci.2014.01.024 24656556 PMC4125475

[2] Gupta RS Warren CM Smith BM Jiang J Blumenstock JA Davis MM Prevalence and severity of food allergies among US adults. JAMA Netw Open. (2019) 2(1):e185630. 10.1001/jamanetworkopen.2018.5630 30646188 PMC6324316

[3] Wang HT Warren CM Gupta RS. Davis CM prevalence and characteristics of shellfish allergy in the pediatric population of the United States. J Allergy Clin Immunol Pract. (2020) 8:1359–70. 10.1016/j.jaip.2019.12.027 31917365 PMC7951995

[4] Gupta RS Springston EE Warrier MR Smith B Kumar R Pongracic J The prevalence, severity, and distribution of childhood food allergy in the United States. Pediatrics. (2011) 128:e9–17. 10.1542/peds.2011-0204 21690110

[5] Sicherer SH Muñoz-Furlong A Sampson HA. Prevalence of seafood allergy in the United States determined by a random telephone survey. J Allergy Clin Immunol. (2004) 114(1):159–65. 10.1016/j.jaci.2004.04.018 15241360

[6] Lao-araya M Trakultivakorn M. Prevalence of food allergy among preschool children in northern Thailand. Pediatr Int. (2012) 54(2):238–43. 10.1111/j.1442-200X.2011.03544.x 22168484

[7] Zeng GQ Luo JY Huang HM Zheng PY Luo WT Wei NL Food allergy and related risk factors in 2540 preschool children: an epidemiological survey in Guangdong Province, Southern China. World J Pediatr. (2015) 11(3):219–25. 10.1007/s12519-015-0030-6 26253412

[8] Li J Ogorodova LM Mahesh PA Wang MH Fedorova OS Leung TF Comparative study of food allergies in children from China, India, and Russia: the EuroPrevall-INCO surveys. J Allergy Clin Immunol Pract. (2020) 8(4):1349–1358.e16. 10.1016/j.jaip.2019.11.042 31857266

[9] Mahesh PA Wong GW Ogorodova L. Prevalence of food sensitization and probable food allergy among adults in India: the EuroPrevall INCO study. Allergy. (2016) 71(7):1010–9. 10.1111/all.12868 27297800

[10] Asero R Antonicelli L Arena A Bommarito L Caruso B Colombo G Causes of food-induced anaphylaxis in Italian adults: a multicentre study. Int Arch Allergy Immunol. (2009) 150:271–7. 10.1159/000222679 19494524

[11] Osterballe M Mortz CG Hansen TK Andersen KE Bindslev-Jensen C. The prevalence of food hypersensitivity in young adults. Pediatr Allergy Immunol. (2009) 20(7):686–92. 10.1111/j.1399-3038.2008.00842.x 19594854

[12] Obeng BB Amoah AS Larbi IA Yazdanbakhsh M van Ree R Boakye DA Food allergy in Ghanaian schoolchildren: data on sensitization and reported food allergy. Int Arch Allergy Immunol. (2011) 155(1):63–73. 10.1159/000318704 21109750

[13] Leung TF Yung E Wong YS Lam CW Wong GW. Parent-reported adverse food reactions in Hong Kong Chinese pre-schoolers: epidemiology, clinical spectrum and risk factors. Pediatr Allergy Immunol. (2009) 20(4):339–46. 10.1111/j.1399-3038.2008.00801.x 18808390

[14] Shek LP Cabrera-Morales EA Soh SE Gerez I Ng PZ Yi FC A population-based questionnaire survey on the prevalence of peanut, tree nut, and shellfish allergy in 2 Asian populations. J Allergy Clin Immunol. (2010) 126(2):324–331.e1-7. 10.1016/j.jaci.2010.06.003 20624649

[15] Yang Z Zhao J Wei N Feng M Xian M Shi X Cockroach is a major cross-reactive allergen source in shrimp-sensitized rural children in Southern China. Allergy. (2018) 73(3):585–92. 10.1111/all.13341 29072879

[16] Greenhawt MJ Singer AM Baptist AP. Food allergy and food allergy attitudes among college students. J Allergy Clin Immunol. (2009) 124(2):323–7. 10.1016/j.jaci.2009.05.028 19560802

[17] Moonesinghe H Mackenzie H Venter C Kilburn S Turner P Weir K Prevalence of fish and shellfish allergy: a systematic review. Ann Allergy Asthma Immunol. (2016) 117(3):264–272.e4. 10.1016/j.anai.2016.07.015 27613460

[18] Nantanee R Suratannon N Chatchatee P. Characteristics and laboratory findings of food-induced anaphylaxis in children: study in an Asian developing country. Int Arch Allergy Immunol. (2022) 183(1):59–67. 10.1159/000518319 34515144

[19] Warren CM Aktas ON Gupta RS Davis CM. Prevalence and characteristics of adult shellfish allergy in the United States. J Allergy Clin Immunol. (2019) 144(5):1435–1438.e5. 10.1016/j.jaci.2019.07.031 31401288 PMC6842441

[20] Woods RK Abramson M Bailey M Walters EH: International prevalences of reported food allergies and intolerances. Comparisons arising from the European Community Respiratory Health Survey (ECRHS) (1991–1994). Eur J Clin Nutr. (2001) 55(4):298–304. 10.1038/sj.ejcn.1601159 11360135

[21] Rahman AMA Helleur RJ Jeebhay MF Lopata AL. Allergic diseases–highlights in the clinic, mechanisms and treatment. Characterization of Seafood Proteins Causing Allergic Diseases. London: IntechOpen (2012).

[22] Rahman AMA Kamath SD Gagné S Lopata AL Helleur R. Comprehensive proteomics approach in characterizing and quantifying allergenic proteins from northern shrimp: toward better occupational asthma prevention. J Proteome Res. (2013) 12:647–56. 10.1021/pr300755p 23268739

[23] Li S Chu KH Wai CYY. Genomics of shrimp allergens and beyond. Genes (Basel). (2023) 14(12):2145. 10.3390/genes14122145 38136967 PMC10742822

[24] Wang J Calatroni A Visness CM Sampson HA. Correlation of specific IgE to shrimp with cockroach and dust mite exposure and sensitization in an inner-city population. J Allergy Clin Immunol. (2011) 128(4):834–7. 10.1016/j.jaci.2011.07.045 21872304 PMC3185202

[25] Uzel A Capan N Canbakan S Yurdakul AS Dursun B. Evaluation of the relationship between cockroach sensitivity and house-dust-mite sensitivity in turkish asthmatic patients. Respir Med. (2005) 99(8):1032–7. 10.1016/j.rmed.2004.12.013 15950145

[26] Wong L Huang CH Lee BW. Shellfish and house dust mite allergies: is the link tropomyosin? Allergy Asthma Immunol Res. (2016) 8(2):101–6. 10.4168/aair.2016.8.2.101 26739402 PMC4713872

[27] Miller JD. The role of dust mites in allergy. Clin Rev Allergy Immunol. (2019) 57(3):312–29. 10.1007/s12016-018-8693-0 29936683

[28] Ruethers T Taki AC Johnston EB Nugraha R Le TTK Kalic T Seafood allergy: a comprehensive review of fish and shellfish allergens. Mol Immunol. (2018) 100:28–57. 10.1016/j.molimm.2018.04.008 29858102

[29] Yu HL Cao MJ Cai QF Weng WY Su WJ Liu GM. Effects of different processing methods on digestibility of Scylla paramamosain allergen (tropomyosin). Food Chem Toxicol. (2011) 49:791–8. 10.1016/j.fct.2010.11.046 21130825

[30] Kamath SD Rahman AMA Voskamp A Komoda T Rolland JM O’Hehir RE Effect of heat processing on antibody reactivity to allergen variants and fragments of black tiger prawn: a comprehensive allergenomic approach. Mol Nutr Food Res. (2014) 58(5):1144–55. 10.1002/mnfr.201300584 24420734

[31] Zhao J Li Y Xu L Ji Y Zeng J Timira V Insight into IgG/IgE binding ability, *in vitro* digestibility and structural changes of shrimp (litopenaeus vannamei) soluble extracts with thermal processing. Food Chem. (2022) 381:132177. 10.1016/j.foodchem.2022.132177 35121318

[32] Faisal M Vasiljevic T Donkor ON. Effects of selected processing treatments on antigenicity of banana prawn (fenneropenaeus Merguiensis) tropomyosin. Int J Food Sci Technol. (2019) 54(1):183–93. 10.1111/ijfs.13922

[33] Santos AB Chapman MD Aalberse RC Vailes LD Ferriani VP Oliver C Cockroach allergens and asthma in Brazil: identification of tropomyosin as a major allergen with potential cross-reactivity with mite and shrimp allergens. J Allergy Clin Immunol. (1999) 104:329–37. 10.1016/S0091-6749(99)70375-1 10452753

[34] Klaewsongkram J. High prevalence of shellfish and house dust mite allergies in Asia-Pacific: probably not just a coincidence. Asian Pac J Allergy Immunol. (2012) 30(4):247–8. 23393903

[35] Matricardi PM Kleine-Tebbe J Hoffmann HJ Valenta R Hilger C Hofmaier S EAACI molecular allergology user’s guide. Pediatr Allergy Immunol. (2016) 27(Suppl 23):1–250. 10.1111/pai.12563 27288833

[36] Ayuso R Reese G Leong-Kee S Plante M Lehrer SB. Molecular basis of arthropod cross-reactivity: igE-binding cross-reactive epitopes of shrimp, house dust mite and cockroach tropomyosins. Int Arch Allergy Immunol. (2002) 129(1):38–48. 10.1159/000065172 12372997

[37] Nugraha R Kamath SD Johnston E Karnaneedi S Ruethers T Lopata AL. Conservation analysis of B-cell allergen epitopes to predict clinical cross-reactivity between shellfish and inhalant invertebrate allergens. Front Immunol. (2019) 10:2676. 10.3389/fimmu.2019.02676 31803189 PMC6877653

[38] Utsch L Logiantara A van Ree R van Rijt LS. Experimental food allergy to peanut enhances the immune response to house dust mite in the airways of mice. Clin Exp Allergy. (2017) 47(1):121–8. 10.1111/cea.12799 27533916

[39] Kulis MD Smeekens JM Immormino M Moran TP. The airway as a route of sensitization to peanut: an update to the dual allergen exposure hypothesis. J Allergy Clin. Immunol. (2021) 148:689–93. 10.1016/j.jaci.2021.05.035 34111450 PMC8429226

[40] Tuano KTS Davis CM. Oral allergy syndrome in shrimp and house dust mite allergies. J Allergy Clin Immunol Pract. (2018) 6(6):2163–4. 10.1016/j.jaip.2018.04.035 29751156

[41] Kanchan K Clay S Irizar H Bunyavanich S Mathias RA. Current insights into the genetics of food allergy. J Allergy Clin. Immunol. (2021) 147:15–28. 10.1016/j.jaci.2020.10.039 33436162 PMC8261836

[42] Sicherer SH Furlong TJ Maes HH Desnick RJ Sampson HA Gelb BD. Genetics of peanut allergy: a twin study. J Allergy Clin Immunol. (2000) 106(1 Pt 1):53–6. 10.1067/mai.2000.108105 10887305

[43] Kivistö JE Clarke A Dery A De Schryver S Shand G Huhtala H Genetic and environmental susceptibility to food allergy in a registry of twins. J Allergy Clin Immunol Pract. (2019) 7(8):2916–8. 10.1016/j.jaip.2019.05.016 31129075

[44] Winters A Bahnson HT Ruczinski I Boorgula MP Malley C Keramati AR The MALT1 locus and peanut avoidance in the risk for peanut allergy. J Allergy Clin Immunol. (2019) 143(6):2326–9. 10.1016/j.jaci.2019.02.016 30825465 PMC6556406

[45] Laha A Ghosh A Moitra S Biswas H Saha NC Bhattacharya S Association of HLA-DQ and IL13 gene variants with challenge-proven shrimp allergy in West Bengal, India. Immunogenetics. (2020) 72(9-10):489–98. 10.1007/s00251-020-01185-3 33175217

[46] Khor SS Morino R Nakazono K Kamitsuji S Akita M Kawajiri M Genome-wide association study of self-reported food reactions in Japanese identifies shrimp and peach specific loci in the HLA-DR/DQ gene region. Sci Rep. (2018) 8(1):1069. 10.1038/s41598-017-18241-w 29348432 PMC5773682

[47] Kılıç M Beyazıt E Önalan EE Kaymaz T Taşkın E. Evaluation of toll-like receptors 2 and 4 polymorphism and intestinal microbiota in children with food allergies. Turk J Pediatr. (2023) 65(5):758–68. 10.24953/turkjped.2023.389 37853967

[48] Rao S Chen X Ou OY Chair SY Chien WT Liu G A positive causal effect of shrimp allergy on Major depressive disorder mediated by allergy- and immune-related pathways in the east Asian population. Nutrients. (2023) 16(1):79. 10.3390/nu16010079 38201909 PMC10780813

[49] Turner AV Smeekens JM. Environmental exposure to foods as a risk factor for food allergy. Curr Allergy Asthma Rep. (2023) 23(8):427–33. 10.1007/s11882-023-01091-0 37227666

[50] Lynch SV Wood RA Boushey H Bacharier LB Bloomberg GR Kattan M Effects of early-life exposure to allergens and bacteria on recurrent wheeze and atopy in urban children. J Allergy Clin Immunol. (2014) 134(3):593–601.e12. 10.1016/j.jaci.2014.04.018 24908147 PMC4151305

[51] Suther C Moore MD Beigelman A Zhou Y. The gut microbiome and the big eight. Nutrients. (2020) 12:3728. 10.3390/nu12123728 33287179 PMC7761723

[52] Fu L Song J Wang C Fu S Wang Y. Bifidobacterium infantis potentially alleviates shrimp tropomyosin-induced allergy by tolerogenic dendritic cell-dependent induction of regulatory T cells and alterations in gut microbiota. Front Immunol. (2017) 8:1536. 10.3389/fimmu.2017.01536 29176981 PMC5686061

[53] Du Toit G Roberts G Sayre PH Bahnson HT Radulovic S Santos AF Randomized trial of peanut consumption in infants at risk for peanut allergy. N Engl J Med. (2015) 372(9):803–13. 10.1056/NEJMoa1414850 25705822 PMC4416404

[54] Sicherer SH Sampson HA. Food allergy: a review and update on epidemiology, pathogenesis, diagnosis, prevention, and management. J Allergy Clin Immunol. (2018) 141:41–58. 10.1016/j.jaci.2017.11.003 29157945

[55] Lack G. Epidemiologic risks for food allergy. J Allergy Clin Immunol. (2008) 121:1331–6. 10.1016/j.jaci.2008.04.032 18539191

[56] Fu L Fu S Wang C Xie M Wang Y. Yogurt-sourced probiotic bacteria alleviate shrimp tropomyosin-induced allergic mucosal disorders, potentially through microbiota and metabolism modifications. Allergol Int. (2019) 68(4):506–14. 10.1016/j.alit.2019.05.013 31262632

[57] Santos AF Riggioni C Agache I Akdis CA Akdis M Alvarez-Perea A EAACI guidelines on the diagnosis of IgE-mediated food allergy. Allergy. (2023) 78(12):3057–76. 10.1111/all.15902 37815205

[58] Gelis S Rueda M Valero A Fernández EA Moran M Fernández-Caldas E. Shellfish allergy: unmet needs in diagnosis and treatment. J Investig Allergol Clin Immunol. (2020) 30(6):409–20. 10.18176/jiaci.0565 32694101

[59] Lieberman JA Sicherer SH. Diagnosis of food allergy: epicutaneous skin tests, *in vitro* tests, and oral food challenge. Curr Allergy Asthma Rep. (2011) 11(1):58–64. 10.1007/s11882-010-0149-4 20922509

[60] Anvari S Brunner S Tuano KS Su BB Karnaneedi S Lopata AL Similar IgE binding patterns in gulf of Mexico and southeast Asian shrimp species in US shrimp allergic patients. Allergy. (2022) 77(9):2825–9. 10.1111/all.15363 35524542 PMC10585665

[61] Mahajan A Youssef LA Cleyrat C Grattan R Lucero SR Mattison CP Allergen valency, dose, and FcεRI occupancy set thresholds for secretory responses to pen a 1 and motivate degn of hypoallergens. J. Immunol. (2017) 198(3):1034–46. 10.4049/jimmunol.1601334 28039304 PMC5263102

[62] Üzülmez Ö Kalic T Breiteneder H. Advances and novel developments in molecular allergology. Allergy. (2020) 75(12):3027–38. 10.1111/all.14579 32882057 PMC7756543

[63] Lee ASE Suprun M Sampson H. Epitope-based IgE assays and their role in providing diagnosis and prognosis of food allergy. J Allergy Clin Immunol Pract. (2023) 11(10):2983–8. 10.1016/j.jaip.2023.06.043 37394177

[64] Wai CYY Leung NYH Leung ASY Ngai SM Pacharn P Yau YS Comprehending the allergen repertoire of shrimp for precision molecular diagnosis of shrimp allergy. Allergy. (2022) 77(10):3041–51. 10.1111/all.15370 35567339 PMC9795902

[65] Conway AE Golden DBK Brough HA Santos AF Shaker MS. Serologic measurements for peanut allergy: predicting clinical severity is complex. Ann Allergy Asthma Immunol. (2024) 132(6):686–93. 10.1016/j.anai.2024.01.018 38272114

[66] Sampson HA Gerth van Wijk R Bindslev-Jensen C Sicherer S Teuber SS Burks AW Standardizing double-blind, placebo-controlled oral food challenges: American Academy of Allergy, Asthma & Immunology-European Academy of Allergy and Clinical Immunology PRACTALL consensus report. J Allergy Clin Immunol. (2012) 130(6):1260–74. 10.1016/j.jaci.2012.10.017 23195525

[67] Santos AF Douiri A Bécares N Wu SY Stephens A Radulovic S Basophil activation test discriminates between allergy and tolerance in peanut-sensitized children. J Allergy Clin Immunol. (2014) 134(3):645–52. 10.1016/j.jaci.2014.04.039 25065721 PMC4164910

[68] Wai CYY Leung NYH Leung ASY Shum Y Leung PSC Chu KH Cell-based functional IgE assays are superior to conventional allergy tests for shrimp allergy diagnosis. J Allergy Clin Immunol Pract. (2021) 9(1):236–44.e9. 10.1016/j.jaip.2020.08.057 32931950

[69] Baek JH Shin YH Chung IH Kim HJ Yoo EG Yoon JW The link between serum vitamin D level, sensitization to food allergens, and the severity of atopic dermatitis in infancy. J Pediatr. (2014) 165(4):849–54.e1. 10.1016/j.jpeds.2014.06.058 25108543

[70] Smeekens JM Kesselring JR Bagley K Kulis MD. A mouse model of shrimp allergy with cross-reactivity to crab and lobster. Methods Mol Biol. (2024) 2717:311–9. 10.1007/978-1-0716-3453-0_21 37737994 PMC11328323

[71] Wai CY Leung NY Ho MH Gershwin LJ Shu SA Leung PS Immunization with hypoallergens of shrimp allergen tropomyosin inhibits shrimp tropomyosin specific IgE reactivity. PLoS One. (2014) 9(11):e111649. 10.1371/journal.pone.0111649 25365343 PMC4218792

[72] NIAID-Sponsored Expert Panel; Boyce JA Assa'ad A Burks AW Jones SM Sampson HA Guidelines for the diagnosis and management of food allergy in the United States: report of the NIAID-sponsored expert panel. J Allergy Clin Immunol. (2010) 126(6 Suppl):S1–58. 10.1016/j.jaci.2010.10.007 21134576 PMC4241964

[73] Nguyen DI Sindher SB Chinthrajah RS Nadeau K Davis CM. Shrimp-allergic patients in a multi-food oral immunotherapy trial. Pediatr Allergy Immunol. (2022) 33(1):e13679. 10.1111/pai.13679 34655480 PMC9297938

[74] Schoos AM Chan ES Wong T Erdle SC Chomyn A Soller L Bypassing the build-up phase for oral immunotherapy in shrimp-allergic children. World Allergy Organ J. (2024) 17(2):100865. 10.1016/j.waojou.2023.100865 38351903 PMC10862060

[75] Theodoropoulou LM Cullen NA. Sublingual immunotherapy for allergy to shrimp: the nine-year clinical experience of a midwest allergy-immunology practice. Allergy Asthma Clin Immunol. (2024) 20(1):33. 10.1186/s13223-024-00895-7 38734651 PMC11088126

[76] Refaat MM Attia MF Saber HM. Desensitization efficacy by sublingual immunotherapy of shrimps extract in asthmatic, rhinitis and Urticaria allergic patients. Food Nutr Sci. (2014) 5:1704–10. 10.4236/fns.2014.517183

[77] Davydov L. Omalizumab (Xolair) for treatment of asthma. Am Fam Physician. (January 2005) 71(2):341–2. 15686303

[78] Patel N Vazquez-Ortiz M Turner PJ. Risk factors for adverse reactions during OIT. Curr Treat Options Allergy. (2019) 6(2):164–74. 10.1007/s40521-019-00205-2

[79] Ebina-Shibuya R Leonard WJ. Role of thymic stromal lymphopoietin in allergy and beyond. Nat Rev Immunol. (2023) 23:24–37. 10.1038/s41577-022-00735-y 35650271 PMC9157039

[80] Chinthrajah S Cao S Liu C Lyu SC Sindher SB Long A Phase 2a randomized, placebo-controlled study of anti-IL-33 in peanut allergy. JCI Insight. (2019) 4(22):e131347. 10.1172/jci.insight.131347 31723064 PMC6948865

[81] Del Rosso JQ. MONOCLONAL ANTIBODY THERAPIES for atopic dermatitis: where are we now in the spectrum of disease management? J Clin Aesthet Dermatol. (2019) 12(2):39–41. 30881583 PMC6415707

[82] Jing ZZ. The Shang Han Lun (Treatise on Cold-Induced Disorders). Written approximately 200 CE).

[83] Bensky D Gamble A. Materia Medica. Seattle: Eastland Press (1993).

[84] Wen MC Wei CH Hu ZQ Srivastava K Ko J Xi ST Efficacy and tolerability of anti-asthma herbal medicine intervention in adult patients with moderate-severe allergic asthma 23. J Allergy Clin Immunol. (2005) 116(3):517–24. 10.1016/j.jaci.2005.05.029 16159618

[85] Li XM Zhang TF Huang CK Srivastava K Teper AA Zhang L Food allergy herbal formula-1 (FAHF-1) blocks peanut-induced anaphylaxis in a murine model. J Allergy Clin Immunol. (2001) 108(4):639–46. 10.1067/mai.2001.118787 11590394

[86] Jing W Liu Q Wang W. Bifidobacterium bifidum TMC3115 ameliorates milk protein allergy in (infants) by affecting gut microbiota: a randomized double-blind control trial. J Food Biochem. (2020) 44(11):e13489. 10.1111/jfbc.13489 32996156

[87] Kubo K Takeda S Uchida M Maeda M Endo N Sugahara S Lit-LAMP-DNA-vaccine for shrimp allergy prevents anaphylactic symptoms in a murine model. Int Immunopharmacol. (2022) 113:109394. 10.1016/j.intimp.2022.109394 36334369

[88] Nunes IV Andrade CM Guerra PV Khouri MI Galantini MPL da Silva RAA A new experimental model to study shrimp allergy. Immunol Lett. (2023) 260:73–80. 10.1016/j.imlet.2023.06.007 37315848

[89] Fang L Zhou F Wu F Yan Y He Z Yuan X A mouse allergic asthma model induced by shrimp tropomyosin. Int Immunopharmacol. (2021) 91:107289. 10.1016/j.intimp.2020.107289 33370683

[90] Wai CY Leung NY Leung PS Chu KH. T cell epitope immunotherapy ameliorates allergic responses in a murine model of shrimp allergy. Clin Exp Allergy. (2016) 46(3):491–503. 10.1111/cea.12684 26610061

